# Medicinal Cannabis and the Intestinal Microbiome

**DOI:** 10.3390/ph17121702

**Published:** 2024-12-17

**Authors:** Luis Vitetta, Tamara Nation, Debbie Oldfield, Michael Thomsen

**Affiliations:** 1 Faculty of Medicine and Health, The University of Sydney, Sydney 2006, Australia; michael@michaelthomsen.com.au; 2 National Institute of Integrative Medicine, Melbourne 3122, Australia; tnation@niim.com.au (T.N.); debbie@healthhouse.com.au (D.O.); 3 Health House Wellness Clinic, Perth 6009, Australia

**Keywords:** medicinal cannabis, exogenous cannabinoids, intestinal microbiota, endocannabinoid system, endogenous endocannabinoids

## Abstract

Historically, the multiple uses of cannabis as a medicine, food, and for recreational purposes as a psychoactive drug span several centuries. The various components of the plant (i.e., seeds, roots, leaves and flowers) have been utilized to alleviate symptoms of inflammation and pain (e.g., osteoarthritis, rheumatoid arthritis), mood disorders such as anxiety, and intestinal problems such as nausea, vomiting, abdominal pain and diarrhea. It has been established that the intestinal microbiota progresses neurological, endocrine, and immunological network effects through the gut–microbiota–brain axis, serving as a bilateral communication pathway between the central and enteric nervous systems. An expanding body of clinical evidence emphasizes that the endocannabinoid system has a fundamental connection in regulating immune responses. This is exemplified by its pivotal role in intestinal metabolic and immunity equilibrium and intestinal barrier integrity. This neuromodulator system responds to internal and external environmental signals while also serving as a homeostatic effector system, participating in a reciprocal association with the intestinal microbiota. We advance an exogenous cannabinoid–intestinal microbiota–endocannabinoid system axis potentiated by the intestinal microbiome and medicinal cannabinoids supporting the mechanism of action of the endocannabinoid system. An integrative medicine model of patient care is advanced that may provide patients with beneficial health outcomes when prescribed medicinal cannabis.

## 1. Introduction

The intestinal microbiome represents a fundamental part of human physiology and metabolic homeostasis, with links that drive health as well as disease [1]. The microbiota in the gut has two broad operational associations, namely with hematological structures (e.g., mucosal immune system) and non-hematological structures (e.g., the gut epithelial barrier). While the molecular mechanisms for drug metabolism in the gut remain mostly improbable, there is increasing evidence that the variability of responses observed to pharmaceutical drugs and medicinal cannabinoid molecules is intestinal microbiome-mediated [2,3].

Pharmacomicrobiomic studies that target intestinal bacteria can significantly influence the pharmacological actions of drugs [4]. Pharmacomicrobiomic studies focus on the intestinal microbiota and the biochemical actions of bacteria to biotransform/metabolize pharmaceutical drugs, xenobiotic compounds and cannabinoid molecules [3,5]. Moreover, biochemical reactions that can change the activity and toxicity of exogenous compounds can thereby shape the host’s response to drugs, xenobiotics and cannabinoids [4,5]. The endocannabinoid system (ECS) of receptors that can act as molecular targets has been reported and suggested as a strategic targeting approach that may improve drug delivery [6]. The ECS is an essential neuromodulatory system that comprises a complex intercellular signaling network, with actions that extend from neural development to modulating the tone of mature synaptic plasticity [7,8].

ECS research that has focused on the actions of intestinal bacteria has reported that the functions of the ECS were linked to important neuromodulatory mechanisms in the gut. A recent study showed that during a lipopolysaccharide (LPS)-induced immune challenge, microglial cells and astrocytes produced TNF-α and IL-1β, which, in turn, induced the production of endocannabinoids [8]. Results confirmed that gut bacteria can modulate the intestinal ECS tone through the effects of a bacteria-derived LPS-dependent mechanism [8]. Furthermore, intestinal microbial dysbiosis has been cited as a key factor [9] in studies with natural cannabis products producing exogenous ECS ligands that has been underscored in chronic users of cannabis [10].

Clinically meaningful relevance refers to a therapy to improve how a patient feels, functions and/or survives [11]. Cannabis molecules that are found in the *Cannabis sativa* Linn. plant, such as delta-9-tetrahydrocannabinol (Δ9-THC) [the major psycho-phytoconstituent], delta-8-tetrahydrocannabinol (Δ8-THC), cannabidiol (CBD), cannabinol (CBN) and cannabigerol (CBG), have been investigated and posited to have clinical relevance [12]. In humans, cannabinoid receptors are distributed on the surface of different cell types [13]. Cannabinoid receptors have different binding affinities for exogenous cannabinoids, the sites where endocannabinoids and molecular derivatives of fatty acids can also bind [13]. The emerging literature suggests that there is a tripartite link between the ECS, cannabis use and the gut microbiome that is of clinical relevance [9].

**Figure 1:**
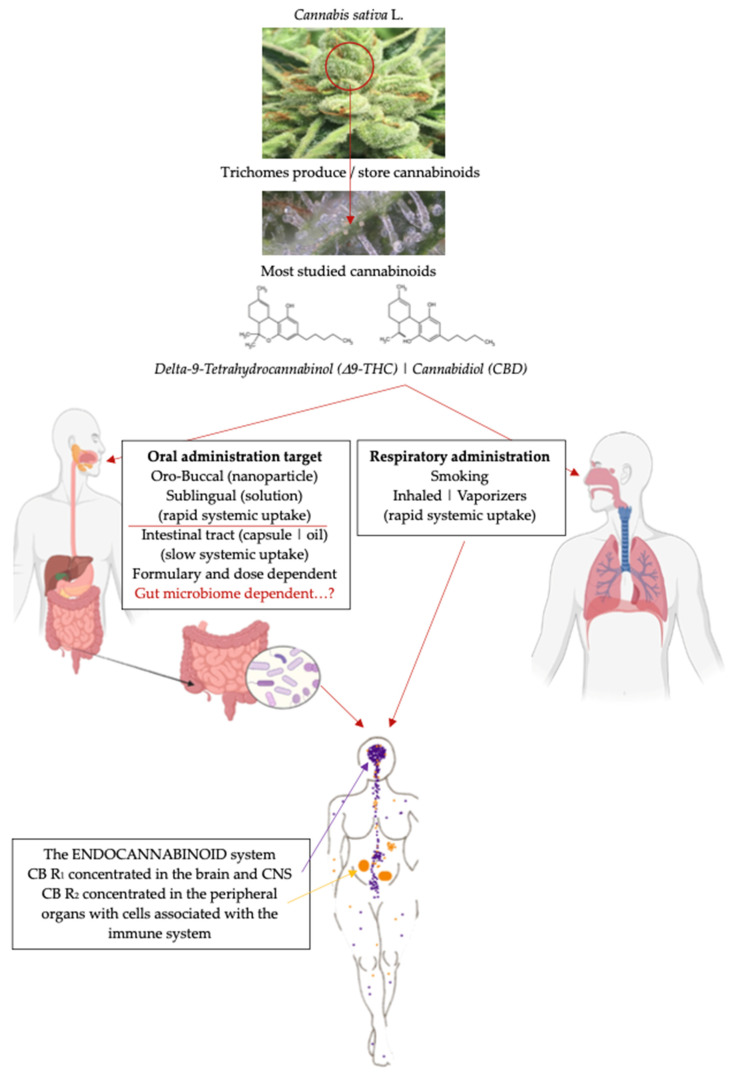
Diverse routes of medicinal cannabis administration [14,15,16,17,18] that target specific organs and the endocannabinoid system influenced by the gut microbiota.

We hypothesize that the intestinal microbiota is an interconnecting axis between the ECS and ingested medicinal cannabis molecules that can then mediate neuro-immunomodulation and progress a crucial positive effect in the regulation of immune and metabolic responses in the gut. If the intestinal microbiome abundance is adversely affected by microbial dysbiosis, medicinal cannabis may be ineffective through the gut, and the effect of medicinal cannabis treatments may be dose-dependent (Figure 1).

## 2. Medicinal Cannabis Administration and the Intestines

The cannabis plant contains a variety of active phytochemicals, including alkaloids, flavonoids, terpenoids, and the cannabinoids (i.e., the latter referred to as containing greater than 100 molecules that are present in the plant) [19,20]. It is reported that the cannabinoids and the receptors that bind these molecules do have a significant effect on the regulation of intestinal peristalsis and intestinal barrier permeability; as such, the cannabinoid molecules show therapeutic potential [21]. Medicinal cannabis can be administered via different routes that include oro-buccal, sublingual and inhaled or smoked paths that have a fast rate of systemic uptake and the oral–intestinal route that has a much slower rate of systemic uptake (Figure 1).

In health, the gut is in a continuous flux of controlled inflammation. The fluidity in the function of the intestines is influenced by the microbiota [22]. The intestinal bacterial cohort generally exists in a symbiosis with the host [22]. This symbiosis importantly directs and assists in stimulating and regulating hematological structures (e.g., mucosal immunity) that maintain immune system equilibrium. In addition, it strengthens non-hematological structures (e.g., the intestinal barrier) that limit the translocation of gut toxins/pathobionts out of the gut lumen [22,23]. The intestinal barrier is a semi-permeable structure that covers the gut and prevents pathogenic molecules and pathobionts from accessing the gut mucosa and systemic circulation that, if breached through intestinal dysbiosis, can progress local inflammatory responses and systemic infections [24,25,26]. Alternatively, the intestinal microbiota is an important participant that allows for nutrient absorption by metabolizing and synthesizing essential vitamins, minerals and amino acids for absorption and eliminating toxic food compounds [26].

Gut microbes can cause a dysbiotic imbalance in the intestinal microbiota [26]. A variety of factors can set this effect in motion, including diets that are high in fat or sugar or low in fiber, the excessive consumption of alcohol daily, consuming produce with elevated levels of pesticide residues [27] or the overprescription of antibiotics [28]. Unbridled intestinal dysbiosis can contribute to and progress, for example, chronic gastrointestinal inflammatory disorders such as irritable bowel syndrome, Crohn’s disease and inflammatory bowel disease [29,30].

A recent review highlighted the effects of cannabinoids on intestinal motility in reducing epithelial gut cell barrier permeability and hence improving gut eubiosis and the therapeutic potential that these molecules may have on intestinal inflammation [21]. In vitro studies have shown that cannabis molecules can elicit a broad antimicrobial activity against a range of microorganisms [31]. Murine experiments with obese mice that were administered Δ9-THC showed that the animals on an obesity-induced feeding diet retained their lean microbiome and avoided becoming obese [32]. CBD, however, did not produce the same effect. The study proposed that THC ameliorated diet-induced obesity and metabolic parameters of low-grade inflammation and reduced intestinal permeability through a mechanism of adipose tissue adaptation [32]. Further, Le Foll and colleagues [33] have reported clinical observations of the prevalence of obesity that was surprisingly much lower in cannabis users as compared to non-users [33]. The difference was not accounted for by tobacco smoking status and was still present after adjusting for variables such as sex and age [33]. However, a study that administered croton oil to induce an inflammatory response in the gut of mice showed that the phytocannabinoid cannabichromene (CBC) normalized in vivo inflammatory hypermotility in the treated mice but did not have any effect on motility in the control group of animals [34].

Consistent with laboratory in vivo reports, the ECS regulates numerous physiological functions in the intestines, including gut motility secretion and visceral sensation. In the intestines, the activation of CB_1_ receptors in the enteric nervous system can be inhibitory for neurotransmitter release, negatively affecting gastrointestinal motility [34] and intestinal secretions [35]. CB_2_ receptor activation in immune cells within the intestinal mucosa can influence inflammatory responses and contribute to intestinal immune equilibrium [36]. Numerous clinical studies show the synthesis and degradation of endocannabinoids, the location of CB receptors, and cannabinoid mechanisms of action on immune/inflammatory, neuromuscular and sensory functions in the intestines (Table 1) [37].

Although Table 1 presents numerous clinical studies that largely show beneficial effects of medicinal cannabis, the administration of medicinal cannabis products can produce significant known side effects. Side effects from medicinal cannabis treatment from both the administration of CBD and THC-containing products can include sedation and fatigue, vertigo and fever. There are also several gastrointestinal-related side effects, such as moderate nausea and vomiting, decreased or increased appetite, a dry mouth, and diarrhea [57]. Specifically, it has been reported that THC-containing products may acutely impair cognitive function [57] and, as such, should not be prescribed to children or adolescents [58]. Furthermore, products that contain THC should not be prescribed to patients diagnosed with angina or who have a documented history of myocardial infarction. In addition, THC-containing products should not be prescribed to those patients who have a personal or family history of mood disorders or are under the medical care of a psychiatrist for a diagnosis of psychosis.

Reports document that approximately 9% of people who identify as chronic cannabis users will show characteristic symptoms of dependence, and 42% of current users meet the criteria for dependence [59,60,61]. Current disorders of cannabis use are more common among males and younger users [61]. The use of cannabis before the age of 17 years was reported to result in people being 18 times more likely to develop cannabis dependence by age 30 years than their peers who did not use cannabis [61].

## 3. Medicinal Cannabis and the Intestinal Microbiota

The microbiota in the intestines represents a fundamental physiological part of human health and disease that can be attributed to the complement of bacteria, viruses and fungi that inhabit the gut [1].

The human gut of an adult individual contains a profile of bacteria that is divided into seven phyla, namely *Firmicutes* (*Clostridium*, *Lactobacillus*, *Enterococcus*), *Bacteroidetes* (*Bacteroides*), *Actinobacteria* (*Bifidobacterium*), *Proteobacteria* (*E. coli*), *Fusobacteria*, *Verrucomicrobia* and *Cyanobacteria* [22]. Members from the *Firmicutes* and *Bacteroidetes* are the major phyla that have been reported to propagate an aberrant metabolism that favors obesity and colorectal cancer development [62]. Investigators have reported that gut bacteria variations, especially from the *Peptostreptococcaceae*, *Veillonellaceae* and *Akkermansiaceae* families, can produce changes in endogenous cannabinoids [63].

Intestinal microbial metabolites such as short-chain fatty acids (SCFAs) have been reported to interact with the ECS and can, in turn, influence its regulation [64]. SCFAs such as butyrate have anti-inflammatory activity in the gut and can also assist with improving gut barrier permeability [65]. Most SCFAs are produced by members from the *Bifidobacterium*, *Lactobacillus*, *Lachnospiraceae, Blautia*, *Coprococcus*, *Roseburia*, *Facealibacterium* and *Oscillospira* genera. Butyrate provides approximately 70% of the energy requisite of intestinal epithelial cells [65]. This energy demand supports intestinal epithelial cell tight-junction protein formation, induces the production of anti-inflammatory cytokines and inhibits histone deacetylase [65].

Intestinal bacteria have also been reported to metabolize tryptophan, an essential amino acid, giving rise to tryptophan bioactive molecules (e.g., indole and its derivatives) [66]. Several intestinal bacterial species have been reported to metabolize tryptophan to indole derivatives, including *Clostridium sporogenes* [67], *Bacteroides thetaiotaomicron*, *Proteus vulgaris* and *Pseudomonas aeruginosa* [68,69]. Li’s recent review [66] presents a comprehensive overview of gut microbiota species that can derive tryptophan metabolites. Tryptophan metabolites produced by gut bacteria are important for the maintenance of the intestinal barrier function and mucosal integrity [70]. In addition, and importantly, tryptophan metabolites significantly influence the regulation of host mucosal immunity.

### 3.1. Metabolic Transformation of Cannabinoids

There is a complex bidirectional interaction between the gut microbiome and cannabis molecules. Studies with murine models have reported that when the animals were treated with orally delivered THC, the gut microbiome expressed an increased abundance of *A. muciniphila*, resulting in improved gut barrier function and improved metabolic health. Human clinical studies have reported that during cannabis use, there was an increase in the abundance of *Bacteriodes*, resulting in increased gut inflammation and an increase in metabolic disorders [71].

The gut microbiota possesses enzymes such as β-glucuronidase, which can deconjugate glucuronide metabolites of THC, releasing the active form back into circulation [72]. Gut bacteria can metabolize THC into 11-hydroxy-THC (11-OH-THC), which is more potent than THC, and 11-nor-9-carboxy-THC (THC-COOH), which is inactive but used as a marker in drug tests [73]. CBD is metabolized into 7-hydroxy-CBD, a compound with potential anti-inflammatory properties [74].

### 3.2. Activation of Cannabinoid Receptors by Gut Microbiota

The gut microbiota can directly influence the pharmacological activity of cannabinoids through the production of secondary bile acids, which can activate cannabinoid receptors. Secondary bile acids are produced through the gut microbiota-mediated deconjugation of primary bile acids synthesized in the liver and secreted into the intestine [75]. These secondary bile acids can activate cannabinoid receptors, particularly CB1 and CB2, which are crucial for the pharmacological effects of cannabinoids [75]. The modulation of cannabinoid receptor activation also plays a significant role. Alterations in the gut microbiota have been associated with changes in endocannabinoid levels and the expression of cannabinoid receptors in the gut and brain, potentially impacting various physiological processes. Gut bacteria-derived metabolites, including secondary bile acids, can activate cannabinoid receptors, influencing the overall pharmacological response to cannabinoids [76].

### 3.3. Implications for Cancer Treatment

Understanding the influence of gut microbiota on cannabis pharmacology has significant implications for cancer treatment related to enhancing therapeutic effects, modulating the immune response and personalizing the treatment. Gut microbiota-targeted interventions, such as dietary changes, probiotics or prebiotics, may enhance the therapeutic effects of cannabinoids in cancer treatment by modulating the gut microbiota. These interventions could potentiate the antitumor effects of cannabinoids or mitigate potential side effects associated with chronic cannabis use [71]. The gut microbiota can also shape systemic immune responses and influence the immune microenvironment of tumors, potentially modulating the response to cannabinoids in cancer therapy [77]. The variability in gut microbiota composition among individuals presents a challenge in developing universal treatment strategies, emphasizing the need for personalized medicine approaches [71]. Profiling the gut microbiota could guide the selection of appropriate cannabis-based formulations, dosages, and treatment regimens tailored to individual gut microbiota compositions [71].

### 3.4. Antimicrobial and Antibiofilm Potential

The growing challenge of antibiotic resistance, particularly in infections associated with biofilm formation, has renewed interest in the antimicrobial activities of cannabinoids.

Animal models have demonstrated and suggested that cannabis consumption via the oral route can change the abundance of specific bacterial taxa following cannabis exposure [78,79]. The antimicrobial potential of CBD has been recently reviewed, concluding that there is significant potential to advance cannabidiol analogs as a new class of antibiotics [80]. In the review, a significant effect of CBD against Gram-positive bacterial activity was reported, and an expanded range of bacterial pathogens was tested. The antibacterial effect on highly resistant bacterial species, such as *Staphylococcus aureus, Streptococcus pneumoniae* and *Clostridioides difficile* [80], was significant. The reported results showed that CBD had outstanding activity against bacterial biofilms and had little propensity to induce resistance, with topical in vivo efficacy. The mode of primary action of CBD proposed was due to bacterial cell membrane disruption, a selective bactericidal effect of CBD on a subset of Gram-negative bacteria that included *Neisseria gonorrhoeae* [80].

CBD and cannabigerol (CBG) have shown potential in reducing the bacterial burden in infections, such as MRSA, and in preventing biofilm formation. These compounds can penetrate biofilms and act on embedded bacteria, which is significant given the antibiotic resistance often exhibited by biofilm-associated bacteria. Additionally, endocannabinoids like anandamide have been found to inhibit biofilm formation and sensitize drug-resistant bacteria to antibiotics [81].

### 3.5. Intestinal Dysbiosis, Pulmonary Fibrosis and Inflammatory Bowel Disease

CBD has been shown to reverse intestinal microbiota dysbiosis and attenuate experimental pulmonary fibrosis induced by bleomycin in rats. CBD was further shown to regulate *Lachnospiraceae*, *Pseudomonas*, *Clostridia*, *Collinsella*, *Prevotella*, *Eubacterium coprostanoligenes*, *Fusobacterium*, *Ruminococcus* and *Streptococcus*. CBD significantly reduced levels of TNF-α, IL-1β, IL-6, MDA and HYP and increased the expression level of SOD (*p* < 0.05). The effects were mediated via key metabolic pathways, including linoleic acid, glycerol, linolenic acid, and sphingolipid metabolism [82]. Cannabinoids interact with ECS receptors (CB1 and CB2) to potentially alleviate symptoms of IBD. Preclinical studies have shown that cannabinoids can reduce inflammation in rodent models of colitis. However, clinical evidence in humans is limited and primarily based on surveys and small trials. Clinical trials have shown that while cannabis can improve quality of life and relieve symptoms like abdominal pain and diarrhea, it does not significantly reduce inflammation markers [83].

### 3.6. Neuroprotective Potential

The potential neuroprotective action of CBD has been examined in an animal model of Alzheimer’s disease. CBD was shown to improve cognitive function in a senescence-accelerated mouse prone 8 (SAMP8) model as evidenced by the Morris water maze test and increased the hippocampal-activated microglia shift from M1 to M2 (M1 microglia release inflammatory mediators and induce inflammation and neurotoxicity, while M2 microglia release anti-inflammatory mediators and induce anti-inflammatory and neuroprotectivity). CBD elevated levels of *Bacteriodetes* that were associated with a fall in *Firmicutes*, morphologically providing a protective intestinal barrier, which subsequently reduced the leakage of intestinal toxic metabolites. Further, CBD was found to reduce the levels of hippocampal and colon epithelial cell lipopolysaccharide (LPS)—known to be increased in AD—leading to impaired gastrointestinal motility and thereby promoting neuroinflammation and subsequent neuronal death [84]. A THC + CBD combination study mitigated experimental autoimmune encephalomyelitis by altering the gut microbiome [78]. Combination-treated animals had significantly higher levels of short-chain fatty acids, such as butyric, isovaleric and valeric acids, than controls and had elevated levels of *A. muciniphila* [78].

An increased level of secondary bile acids within the brain during neurodegeneration has been reported [85]. The most likely explanation for this effect is a resultant change in the gut microbiome production of higher levels of secondary bile salts, which subsequently enter the brain [85]. Bile acids can form complexes with exogenous cannabinoid molecules [71], which can lead to a slower rate of reabsorption and/or excretion with a subsequent increased rate of retention in the gut, thereby mediating changes in the tone of cannabinoid receptor activation [71].

### 3.7. Cardioprotective Potential

The ability of CBD to reduce cardiovascular risk factors, including trimethylamine-N-oxide (TMAO) and phenylacetylglutamine, has been examined in a mouse model using 16S ribosomal RNA (rRNA) gene sequencing and ultra-high performance liquid chromatography-quadrupole time-of-flight mass spectrometry-based metabolomics. CBD decreased the levels of creatine kinase, alanine transaminase (ALT) and low-density lipoprotein cholesterol and markedly increased high-density lipoprotein cholesterol. CBD treatment increased the abundance of beneficial bacteria, which include Lachnospiraceae_NK4A136 and *Blautia* in the gut and increased the levels of TMAO and phenylacetylglutamine in the plasma [86].

### 3.8. Gut Microbiota Phenotype and Obesity

A high *Firmicutes*-to-*Bacteriodetes* ratio is considered a pro-obesity gut microbiota phenotype associated with the metabolic syndrome [87]. Recent evidence has demonstrated that this pro-obesity skewed ratio can be restored in mice treated with THC [88]. In obese murine models, the intestinal microbiome modifies endocannabinoid signaling [88]. The overall effect is increased gut permeability alongside obesity-associated low-grade inflammation, as well as adipogenesis [88]. THC was demonstrated to prevent the pro-inflammatory effect in mice. *Akkermansia muciniphila* is a commensal gut bacterium whose relative increased abundance has been associated with enhanced intestinal barrier function and metabolic health [78,88]. THC demonstrably increases the abundance of *A. muciniphila,* facilitating weight loss through the control of fat storage and adipose tissue metabolism [88]. Mechanistically, *A. muciniphila* also reduces levels of the pro-inflammatory cytokine IFN-γ, a decrease that is known to improve glucose tolerance with subsequent control of glucose metabolism [88]. Mice fed a high-fat diet with an added cannabis extract resulted in more favorable modifications in the intestinal microbiota [89]. Overall, the study showed that the addition of different cannabis plant strain extracts of distinct CBD/THC profiles to a high-fat diet unsuccessfully mitigated metabolic perturbations. Notwithstanding this, the results also demonstrated that the cannabis extract established a significant positive impact on the intestinal microbiota profile [89]. The overall impression from these studies strongly suggests that oral consumption of cannabis can influence the intestinal microbiota composition, potentially with direct effects on gut health and physiological processes that can be regulated (e.g., mucosal immunity anti-inflammatory responses) by the gut microbiome. Equally, cannabis extract exposures with ineffective formulations could be linked to an increased abundance of *Bacteroides* species in the human intestinal microbiota, which might be associated with gut inflammation and metabolic disorders [79]. The microbiome of cannabis users has been shown to display a *Prevotella*/*Bacteroides* ratio that is 13-fold lower than that of non-users [10].

## 4. Medicinal Cannabis Administration and the Endocannabinoid System

The endocannabinoid system (ECS) regulates and controls various critical physiological functions, from learning/memory/emotional processing to sleep, body temperature control, nutrition and inflammatory and immune responses [90] (Figure 1). The ECS comprises an extensive network of chemical signals linked to cellular receptors that are densely crowded throughout body tissues, as well as in the brain [90]. In the brain, for example, the receptors behave as a central point that controls the levels and activity of other neurotransmitters. Fast-acting feedback loops operate to tune upward or downward the activity of any system that needs adjusting, as would happen, for example, for body temperature control, alertness and inflammatory responses [90].

The endocannabinoid system comprises the endocannabinoid ligands anandamide (AEA) and 2-arachidonoylglycerol (2AG), the enzymes (i.e., N-acylphosphatidylethanolamine-hydrolysing phospholipase D) for the synthesis of AEA, fatty acid amide hydrolase (FAAH) for the degradation of AEA, and monoacylglyceride lipase for the degradation of 2AG. The ECS comprises two main cannabinoid receptors, namely type 1 (CB_1_) and type 2 (CB_2_). There is thought to be a third receptor in the ECS, which is G-protein-coupled receptor 55 (GPR55).

**Figure 2:**
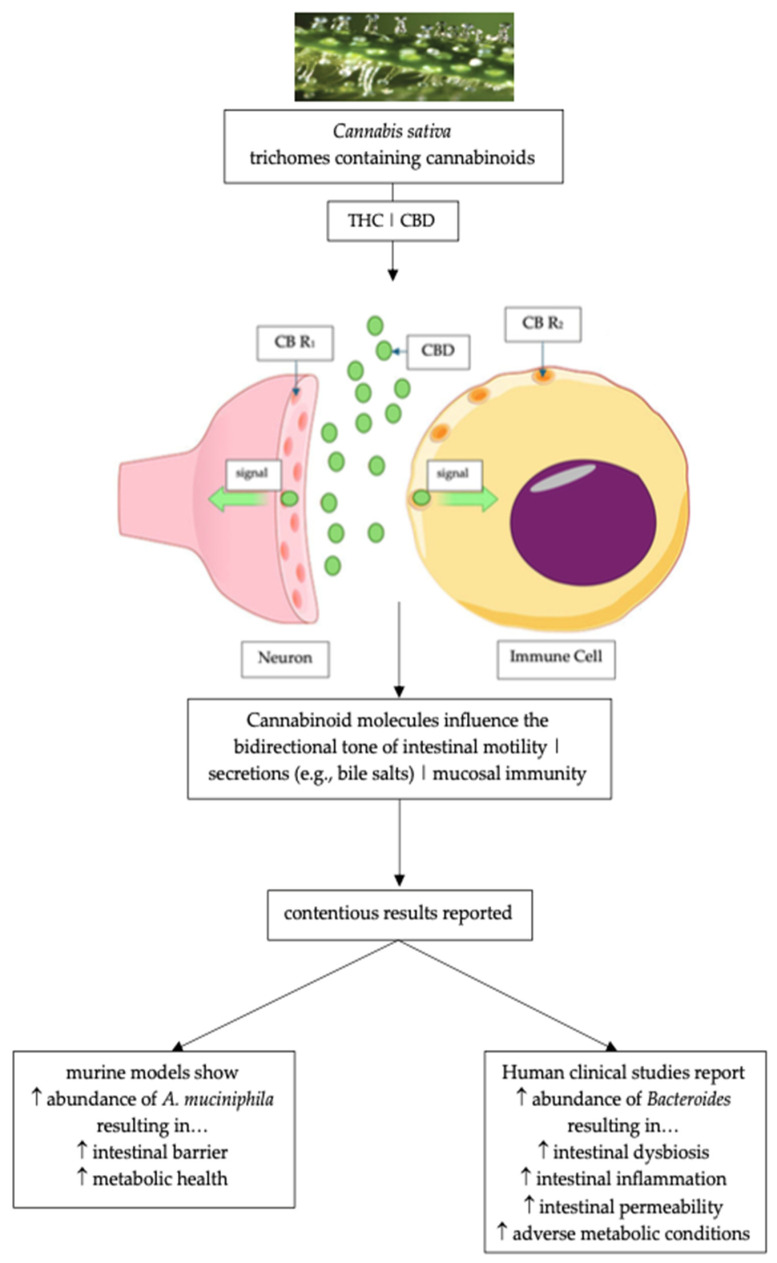
Diagrammatic representation of ECS receptors CB R_1_ and CB R_2_ that have been identified on neuronal cells [95] and immune system cells (i.e., B lymphocytes) [96] and can bind CBD and THC and their effects on the gut and intestinal microbiota (adapted and modified from Izzo and Sharkey; Storr et al.; Al-Khazaleh et al.) [35,36,71]. ECS = endocannabinoid system; CBD = cannabidiol; CB R_1_ = Cannabinoid Receptor 1; CB R_2_ = Cannabinoid Receptor 2.

Biochemically, the endocannabinoid molecules have structural similarities to the molecules from the *Cannabis sativa* Linn. plant (Figure 2). The most frequently studied molecules from the cannabis plant (e.g., THC, CBD) are ligands that can bind to the ECS receptors. It is reported that THC’s actions are largely mediated by strong affinity binding to and activation of CB1 receptors [91,92], whereas CBD binds to CB_1_ and CB_2_ receptors with weak affinity [93]. CBD was reported to behave as a non-competitive negative allosteric modulator of CB_1_ receptors [94]. The importance of the Laprairie et al. study [94] advanced the posit that allosteric modulation in combination with the effects not mediated by CB_1_ receptors may explain the in vivo effects of CBD. The effects of allosteric modulators of CB_1_ receptors of the ECS have the potential to treat CNS and peripheral disorders while sidestepping the adverse effects associated with orthosteric agonism or the antagonism of these ECS receptors [94].

## 5. The Cannabis Receptors and Immune Function in the Intestines

There are numerous receptors in the gastrointestinal tract that can interact with cannabis molecules [97].

ECS receptors have a ubiquitous distribution in humans. CB_1_ receptors are highly expressed in the brain and peripheral tissues, such as in adipocytes, circulating immune cells, skeletal muscle, exocrine pancreas, liver and the gastrointestinal tract [1], whereas CB_2_ receptors are present in the spleen, thymus, pancreas and peripheral immune cells, including mast cells and peripheral blood leukocytes. There are thought to be fewer CB_2_ receptors in the central nervous system than CB_1_ receptors.

Both the CB_1_ and CB_2_ receptors are G-protein-coupled receptors. Their endogenous ligands are the arachidonate-derived molecules N-arachidonylethanolamine (AEA) and 2-arachidonylglycerol (2AG), respectively [1,4]. AEA and 2AG are endogenous lipids that engage the CB_1_ and CB_2_ receptors and have been shown to modulate neurologic activity and human behavior in a mechanism like that of the main psychoactive component of the plant *Cannabis sativa*, THC. Termination of signaling occurs via reuptake, and enzyme hydrolysis occurs primarily by fatty acid amide hydrolase (FAAH) and monacylglyceride lipase [5].

The phytocannabinoid CBD has been reported to affect the tone and hence the activity of many cellular effectors, of which the most common include CB_1_ and CB_2_ receptors [98].

The pharmacology of drug specificity is crucial in drug design and development, a supposition that can also be applied to cannabinoid formulations [99]. This idea is conceptualized from the observed results from numerous in vitro and in vivo studies [100]. These studies show that CBD has receptor-specific effects reported as antagonistic to cannabinoid agonists at CB_1_ and CB_2_ receptors. The effect is reported at concentrations significantly below the affinity for CBD to the orthosteric agonist site of these receptors [21,100,101,102,103]. Furthermore, CBD had negative agonistic actions for both CB_1_ and CB_2_ receptors [21].

The in vitro effects of CBD on intracellular signaling have been reported to be largely independent of CB_1_ receptors [104]. CBD was shown to inhibit the internalization of CB_1_ receptors from in vitro studies at sub-micromolar concentrations, and the study concluded that no other CB_1_ receptor-dependent effect on signaling was observed [104].

Furthermore, CBD has been reported to interact with 5HT_1A_ receptors [105], G-protein-coupled receptor 55 (GPR55) [106], the μ- and δ-opioid receptors [107], the transient receptor potential vanilloid 1 (TRPV1) cation channels [108], peroxisome proliferator-activated receptor gamma (PPARγ) [109] and FAAH [109].

It is known that exogenous cannabinoid ligands interact with the subtype 5HT_1A_ receptor [110]. In addition, interesting murine studies have reported and suggested that CBD can induce antipsychotic-like effects by activating 5-HT1A receptors [111]. This indicates that CBD could be an alternative treatment for negative and cognitive symptoms of schizophrenia [111,112]. CBG has been reported as a moderate 5-HT_1A_ receptor antagonist [113]. In a recent study [114] with rats, CBG hindered the inhibitory effect produced by selective α2-adrenoceptor and 5-HT1A receptor agonists on the firing rate of NA-LC and 5-HT-DRN neurons (i.e., mechanism unknown), producing anxiolytic-like effects through 5HT_1A_ receptors [114].

The GPR55 is a receptor involved in proliferation, differentiation, and cytoskeletal modulation [115]. In experimental genetically induced Dravet syndrome studies, CBD has been reported to elicit anti-inflammatory effects [116], as well as experimental Parkinson’s disease [117,118].

CBD and CBG have been reported to impact nuclear receptors of the PPARγ family [119,120]. Consequently, the activation of PPARs inhibits the transcription of pro-inflammatory genes and cytokines TNF-alpha, IL-1 beta and IL-6, preventing the NF-kappa B signaling pathway [121]. The significance of this inhibitory effect on transcription factor NFκB is that it is a critical trigger in regulating cellular inflammation that can lead to neuronal death, an effect that describes why PPARs have been proposed as possible targets for neuroprotection [120]. In vitro and in vivo experimental studies have reported that CBD [119] had an anti-inflammatory effect elicited through PPARγ in multiple sclerosis [122], ischemic stroke [123] and Parkinson’s disease [124]. CBG has also been reported to exhibit regulated neuroinflammation processes with a dual PPARα/γ agonist effect [125]. The anti-inflammatory effects mediated by PPARs trigger the reduced pro-inflammatory activity of transcription factors, which consequently regulates the expression of genes that are responsible for inflammation [121].

The transient receptor potential vanilloid (TRPV) describes one of the seven sub-families of receptors within the transient receptor potential (TRP) family [126]. These receptors demonstrate activity in the sensation of heat and pain. CBD has been found to display agonistic activity on TRPV 1–4 [127]. The interactions of CBD with TRPV1 could be important for pain relief. Hence, TPRV1 and TPRV4 that are expressed in the intestines may represent targets for IBD [128], given that they exert pro-inflammatory effects in the intestines. The overall effects of the in vivo involvement of these molecular targets in the actions of CBD in the intestines remain to be elucidated.

## 6. Discussion

With the increased legalization of medicinal cannabis and the marketing of CBD-rich products, there is renewed interest in the treatment of anxiety, nausea, sleep disturbances, joint pain and other common complaints. Consequently, the molecules from the *Cannabis sativa* Linn. plant or hemp-derived CBD have been employed in clinical studies investigating inflammatory and metabolic factors to improve health outcomes.

Laboratory and clinical investigations acknowledge and recognize a tripartite interaction between the gut microbiome, the ECS and medicinal cannabis molecules that are mostly orally administered. The gut microbiome comprises a complex ecosystem that manipulates fundamental physiological processes, including nutrients, pharmaceutical drug and neuroendocrine metabolism, neuro-immune regulation and protection from pathogenic agents. Since the inception of the human microbiome project, a number of gut axes have been progressed and investigated [129]. This further emphasizes the scientific evidence that links the intestinal microbiome to end-organ functions outside of the gut. Accordingly, medicinal cannabis is postulated to improve symptom management in different anatomical areas.

Gut microbial dysbiosis has been associated with numerous host health conditions [130]. Diseases that have been correlated with intestinal microbial imbalances include inflammatory bowel disease, obesity, cancer and neurodegenerative disorders [131]. Metabolome analytical studies can determine important metabolites with biological implications in host–gut microbiota interactions. The study of metabolomics will continue to target small molecule metabolites (e.g., SCFAs) that impact the host metabolome, focusing on biochemical functions that have shown promise for studying host–gut microbiota interactions. A multi-omic analysis study of the microbiome and metabolome relevant to dietary intake identified dietary compounds and phytochemicals [132]. These compounds were postulated to regulate the tone of gut bacterial abundances within the intestines and interact with the microbiome composition to alter favorably host metabolism [132].

Intestinal microbiome profiles can be distinctive in different individuals at the species and genus levels, which has been suggested to be dependent on lifestyle factors intertwined with an individual’s genetic make-up, nutritional choices, environmental conditions, early microbial exposure post-birth and the achievement of metabolic and immunological equilibrium in early life [130,133,134]. Deviations from the normal composition and diversity of the intestinal microbiome, characterized by an imbalance and decreased abundance eliciting functional changes in the ecology of the microbial cohort, progresses intestinal dysbiosis. Gut microbial dysbiosis could disrupt medicinal cannabis efficacy.

Intestinal bacteria are known to significantly influence the metabolism and pharmacokinetic actions of synthetic and naturally derived drugs [135], including cannabis molecules. Preclinical studies report that the intestinal microbiota can significantly influence the pharmacological effects of exogenous cannabinoids [71]. While clinical studies report that orally ingested medicinal cannabis formulations can positively influence gut motility and anti-inflammatory sequelae in the gut [136], studies have also reported no effects with CBD oil in the range of 50 to 600 mg/day for 4 weeks [53] and CBD oil at a dose of 18–21 g/day for 24 weeks [56]. The presence of intestinal microbial dysbiosis may be a gastrointestinal tract factor that impacts cannabis activity and efficacy.

A preliminary study with chronic cannabis users reported that marijuana use was associated with adverse shifts in the gut microbiota, suggesting that marijuana-associated low vegetable and fruit intake may have contributed to the microbiome changes observed [10]. Additionally, the intricate link that exists between the gut microbiota and the ECS could be influenced by abundance changes in the gut microbiota, prompting adverse ECS tone and destabilizing the intestinal barrier integrity. These adverse changes suggest that microbial dysbiosis can be the cause of the ambiguous role of the ECS in health and disease (e.g., obesity) [137].

Preclinical and clinical studies have provided evidence that adverse changes in gut–brain interactions that can progress to depressive disorders begin in a dysbiotic gut [138]. How microbial species in the gut can shape the features of brain functioning (e.g., memory, social behavior) and how gut microbial species may be contributing to conditions such as anxiety, depression and neurodegenerative diseases largely remain to be further elucidated. There is a complexity of interconnections that exist in the gut that are supported by ideas that posit the actions of multiple biochemical components that underpin the microbiota–gut–brain axis [138]. Recently, it was reported that with the specific use, prescribed or otherwise, of exogenous cannabis, epigenetic changes were induced that modified the tone of depressive-anxious, psychotic and addictive behavioral phenotypes [139]. In the presence of reduced gut microbiome abundance, which describes intestinal microbial dysbiosis, a clinical link is established with adverse cognitive function and behavior [140]. The long-term use of medicinal cannabis can further adversely affect cognitive activity/ability, exacerbating the emergence of psychotic symptoms and prolonging recovery.

Providing patient-centered care underpins the modalities of integrative medicine. The combination of a conventional medical approach with an integrative model of care can provide a benefit with effective treatments. The employment of cannabis as an adjunct to standard medical therapy for conditions such as nausea, anxiety, joint pain and gut health may complement and facilitate improvements in patient health outcomes.

## 7. Conclusions

The current clinical evidence from human studies of orally administered medicinal cannabis formulations (e.g., CBD products) is skewed towards high doses, indicating that in the gut, efficacy may be dose-dependent. In humans, there is evidence to suggest that cannabis affects the microbiome. The demonstrated modulatory effects on the gut are on both the intestinal barrier permeability and the intestinal microbiome abundance. Intestinal microbial dysbiosis is a characteristic that describes an altered gut microbiome ‘richness’ in combination with the physiological activity of the ECS; these are important factors that determine cannabis oral administration efficacy. Therefore, in some patients who request a medicinal cannabis prescription, a medical assessment of gut issues (e.g., constipation, abdominal pain) may be necessary to support an integrative medical approach that targets and recovers the microbial dysbiotic gut prior to the use of medicinal cannabis.

Furthermore, other modes of delivery of medicinal cannabis, such as oro-buccal, sublingual and inhaled/smoked alternatives, provide cannabinoids that have rapid access to the systemic circulation, bypassing the intestinal tract. Caution is always indicated when any medicinal cannabis product is prescribed by starting with a ‘low dose’ and titrating up when medically indicated.

## Figures and Tables

**Table 1:** Human clinical studies investigating cannabinoid effects on gut metabolism and immune/inflammation.

Cannabis Source * (n = Participants) [Reference]	Study Type and Condition	Dose Administered	Metabolic|Immune|Inflammation Effects
Cannabis effects specific to the intestines			
Δ9-THC from flowers Source: *Cannabis sativa* (21) [38]	RCT Active CD	—Dose: 115 mg/8 weeks	—Clinical response 10/11 vs. 4/10 in placebo group (*p* = 0.028) —Complete remission in 5/11
Oral Δ9-THC Synthetic source (17) [39]	Single-blinded, PCT [2 × 2] crossover trial —food stimuli|intake —metabolic hormone responses	Four groups Dronabinol capsules 2.5 mg Δ^9^-THC depending on BW dose range of 5 to 10 mg| —THC/oral intake —THC/intragastric infusion —placebo/oral intake —placebo/intragastric infusion	Compared with placebo THC users: ↑ liking of high-calorie images (*p* = 0.0031) ↑ wanting of high-calorie images (*p* = 0.0096) ↑ milkshake consumption (*p* = 0.0005) but not intragastric ↑ prospective food consumption (*p* = 0.0039) Prior to milkshake consumption, THC ↑ plasma motilin (*p* = 0.0021); ↓ octanoylated ghrelin (*p* = 0.023); ↓ glucagon-like peptide 1 response during both oral (*p* = 0.0002) and intragastric (*p* = 0.0055). —Overall findings suggest ECS drives food intake by interfering with anticipatory|cephalic phase|metabolic hormone responses.
CBD and PEA Source: *Cannabis sativa* (30) males only [40]	DBPCRCT —Healthy individuals —Aspirin dose 600 mg oral +400 mL water induced ↑ gut permeability	Participants treated with PEA oral dose 600 mg CBD oral dose 600 mg or placebo In addition: —1 g of lactulose —1 g of mannitol in 600 mL water administered	—Aspirin caused ↑ absorption of lactulose ↑ absorption of mannitol —Which was ↓ PEA or CBD (*p* = 0.001) —Overall result: CBD and PEA can reduce permeability in the human colon.
CBD Chewing gum Source not available (32) females only [41]	DBPCRCT cross-over study —IBS with abdominal pain	—Chewing gum formulation containing 50 mg CBD or placebo. —8 weeks.	—No differences at the group level were reported between CBD and placebo gum in pain scores or the number of gums used
CBD Pharmaceutical grade|source not specified. (48) [42]	DBPCRCT Parallel group —Patients with FD non-delayed GE	Dose —CBD b.i.d. (20 mg/kg/d) —4 weeks	CBD and placebo effects on physiological functions and patient response outcomes were not significantly different. Borderline CBD treatment-by-genotype interactions: —rs806378 CNR1 with Leuven Postprandial Distress Scale (*p* = 0.06). —GE solids (*p* = 0.12).
Oral Δ9-THC Synthetic source (36) [43]	DBRCT —IBS–D Assessments —gastric transit —small bowel transit —colonic transit genotyped SNPs —CNR1 —rs806378—FAAH —rs324420	Three group allocations: Dronabinol —2.5 mg/2 days —5 mg/2 days —placebo/2 days	No treatment effects on gastric|small bowel|colonic transit. —Genotypes CNR1|rs806378|CT/TT associated with modest delay in colonic transit at 24 h compared with CC (*p* = 0.13) genotype. —Overall result: Dronabinol a nonselective cannabinoid receptor agonist does not significantly affect colonic transit. Dronabinol may inhibit colonic transit in a subset of IBS-D patients based on a specific genetic variation in CB_1_.
CBD Source: epidiolex Jazz Pharmaceuticals (44) [44]	Patients with nonsurgical gastroparesis with delayed gastric emptying of solids. —32 idiopathic —6 DMT1 —6 DMT2	Administered epidiolex dosing regimen: —orally twice daily in equally divided doses starting at 2.5 mg/kg/d, increasing by 2.5 to 5.0 mg/kg every other day until the target dose of 20 mg/kg/d was reached. —4 weeks.	CBD significantly ↓ the total Gastroparesis Cardinal Symptom Index score (*p* = 0.008); ↓ inability to finish a normal-sized meal (*p* = 0.029); ↓ number of vomiting episodes/24 h (*p* = 0.006); ↓ overall symptom severity (*p* = 0.034). Patients treated with CBD ↑ volume to comfortable fullness and maximum tolerance and slower GE. —FAAH|rs34420 genotype significantly impacted nutrient drink ingestion. —Overall result: CBD provided symptom relief in patients with gastroparesis|improved tolerance of liquid nutrient intake|despite slowing GE.
CBD Source: *Cannabis sativa* (48) [45]	Adult patients undergoing alloHCT	Administered CBD at 300 mg/day for 7 days prior to transplantation. Continued until day 30. —30 days CBD treatment.	None of the patients developed acute GVHD while consuming CBD. —Patients surviving >100 days Cumulative incidences of moderate-to-severe chronic GVHD at 12 and 18 months were 20% and 33%, respectively. —Overall result: combination CBD+ standard GVHD prophylaxis was a safe and promising strategy to reduce the incidence of acute GVHD.
Oral Δ9-THC Source: *Cannabis sativa* (13) [46]	DBRCT —Effect on gastric emptying of solid foods in humans.	Oral administered —dose: THC 10 mg/m^2^ of body surface area or placebo —2 days	—No correlation was found between plasma THC levels and the delay in gastric emptying. —THC at a dose used for preventing chemotherapy-induced nausea and vomiting significantly delays gastric emptying of solid food in humans.
THC Source: dried flowers of genetically identical plants of *Cannabis sativa* var. Indica “Erez” (courtesy of Tikun-Olam Ltd., Tel Aviv, Israel) (32) [47]	DBPCRCT —patients with UC	Smoking administered —dose: 80 mg 16% THC —additional content: 0.5% CBG 0.1% CBD traces < 0.1% CBC|CBDV|Δ8THC —8 weeks	—Short-term treatment with THC-rich cannabis-induced clinical remission improved quality of life in patients with mild to moderately active UC. —Beneficial clinical effects were not associated with significant anti-inflammatory improvement.
CBD-rich botanical extract (60) [48]	DBPCRCT parallel allocation —patients with mild to moderate UC	Oral administration —50 mg CBD-rich extract versus placebo (1:1) b.i.d. —12 weeks	—Primary endpoint was negative —End-of-treatment remission rates were similar for CBD-rich botanical extract (28%) and placebo (26%). Per Protocol analysis of total and partial Mayo scores favored —CBD-rich botanical extract (*p* = 0.068 and *p* = 0.038, respectively. Per Protocol analyses of the more subjective physician’s global assessment of illness severity, subject global impression of change, and patient-reported quality-of-life outcomes —improved for CBD-rich botanical extract (*p* = 0.069, *p* = 0.003, *p* = 0.065, respectively).
Cannabis effects specific to gut immunity/inflammation			
Δ^9^-THC Synthetic source (Marinol) (100) [49]	PCRCT —Patients with stable MS —Serum cytokine levels of IFN-γ IL-10|IL-12	Oral administered —dose: 0.25 mg/kg/day of Δ^9^-THC —matched placebo —13 weeks	—no evidence for cannabinoid influence on serum levels of interferon (IFN)-γ interleukin (IL)-10, IL-12 or C-reactive protein
CBD|THCV Cannabinoids source not specified (62) [50]	Pilot DBRCT Non-insulin-treated T2DM and dyslipidemia	Three group assignments: —1:1 ratio of CBD 5 mg and THCV 5 mg|b.i.d. —20:1 ratio of CBD 100 mg THCV 5 mg, b.i.d. —matched placebo —13 weeks	Compared to placebo THCV ↓ FPG (*p* < 0.05) —improved pancreatic b-cell function (HOMA2 b-cell function) (*p* = 0.01) —improved adiponectin (*p* = 0.01) —improved adiponectin A (*p* < 0.05) —plasma HDL, no effect
CBD Source: purified, hemp-derived (28) [51]	DBPCRCT Assessments —mental health —sleep quantity and quality —immune cell function —healthy college-aged individuals.	Two group allocations: —CBD group 50 mg —calorie-matched placebo group —8 weeks	No significant differences between CBD and CBN groups on mental health measures, sleep quantity, circulating immune phenotype. —CBD group significant improvements: sleep quality (*p* = 0.0023); enhanced natural killer immune cell function (*p* = 0.0125).
CBD oil Hemp-derived Source: SunFlora Inc. (3) PLWH [52]	Analyzed ~41,000 human PBMCs from three PLWH: —baseline —after CBD treatment —27–60 days —through single-cell RNA sequencing.	—CBD-rich formulation (~4–7%) containing THC (<0.3%) and all naturally occurring cannabinoids|terpenes|essential oils from the plant extract. —minimum 4 weeks of treatment	—CBD associated with alterations of gene expression in myeloid cells after treatment.
CBD oil Source unknown (81) [53]	RCT —CBD anti-inflammatory effect in advanced cancer —CRP —inflammatory cytokines —IFN-gamma| —Hu IFN-a2| Hu IL-8 Hu IL-10 2 20 26 32 34 or 35 Hu MMP-1 Hu LIGHT.	Randomized —escalating doses of CBD oil (range 50 to 600 mg/day) or placebo oil —4 weeks	—No difference between the two arms in the trajectory of CRP or cytokine levels from baseline to day 28 (4 weeks).
CBD Source undisclosed Concomitant use of cocaine (81) males [54]	Peripheral blood assays for the following: —sCD14|LPS —inflammatory TNF-α|IL-6| —regulatory IL-10 cytokines|CRP|TBARS for lipid peroxidation|total thiols	Three GROUPS —cannabis (n = 21) —cocaine (n = 12) —cannabis + cocaine (n = 27) —non-drug users (n = 21) —current use	Cannabis contributes to an anti-inflammatory/or regulatory profile. —Concomitant cannabis + cocaine consumption coexists with ↑ circulating LPS and pro-inflammatory status.
THC Source: pharmaceutical quality (18) healthy females [55]	DBPCRCT cross-over —healthy females	Sunburn spot was induced on one upper leg —dose: 20 mg THC + CBD 2:1 ratio|other plant cannabinoids <5% in the capsule. —8 hr treatment	—Cannabis extract did not affect heat pain thresholds in the sunburn model. —No analgesic or anti-hyperalgesic activity of the cannabis extract was reported.
Hemp Source undisclosed (100) relapsing MS [56]	—EDSS —serum levels of liver enzymes GGT|AST|ALT	DBRCT Three GROUPS —A hemp + evening primrose oil + hot nature diet|dose 18–21 g/day (6–7 g t.i.d.) —B olive oil|dose 18–21 g/day (6–7 g t.i.d). —CBD co-supplemented oils|dose 18–21 g/day (6–7 g t.i.d.) —24 weeks	—No significant difference in the study parameters at baseline. In groups A and C after intervention: —↓ EDSS score significantly —↓ levels of liver enzymes —↑ serum liver enzymes and EDSS score in group B.

* THC = Δ9-tetrahydrocannabinol; THCV = D9-tetrahydrocannabivarin; CBD = cannabidiol; CD = Crohn’s disease; b.i.d. = twice per day; FPG = fasting plasma glucose; HDL = high-density lipoprotein; PCT = placebo-controlled trial; BW = body weight; ECS = endocannabinoid system; DBPCRCT = double-blind placebo-controlled randomized clinical trial; DBRCT = double-blind randomized clinical trial; PEA = palmitoylethanolamide; IBS = irritable bowel syndrome; IBS–D = irritable bowel syndrome with diarrhea; FD = functional dyspepsia; GE = gastric emptying; SNPs = single-nucleotide polymorphisms; GVHD = graft-versus-host disease; AlloHCT = allogeneic hematopoietic cell transplantation; DMT1 = diabetes mellitus type 1; DMT2 = diabetes mellitus type 2; MS = multiple sclerosis; IFN-γ = interferon-gamma; IL-10 = interleukin-10; -12; UC = ulcerative colitis; PLWH = people living with HIV; PBMCs = peripheral blood mononuclear cells; sCD14 = soluble CD14; TBARS = thiobarbituric acid-reactive substance; t.i.d. = three times per day; GGT = gamma-glutamyl transferase; AST = aspartate aminotransferase; ALT = alanine aminotransferase.

## Data Availability

Not applicable.
